# *Lactobacillus crispatus* Strain KT-11 S-Layer Protein Inhibits Rotavirus Infection

**DOI:** 10.3389/fmicb.2022.783879

**Published:** 2022-02-22

**Authors:** Takeshi Kawahara, Issei Shimizu, Yuuki Tanaka, Keisuke Tobita, Mikado Tomokiyo, Itsuki Watanabe

**Affiliations:** ^1^Faculty of Agriculture, Shinshu University, Nagano, Japan; ^2^KITII Co., Ltd., Tokyo, Japan

**Keywords:** *Lactobacillus crispatus*, S-layer protein, lithium chloride extract, rotavirus, infection

## Abstract

S-layer proteins (SLPs), which are present in the external layer of certain strains of lactic acid bacteria isolated from the intestinal tract, are known to recognize and bind to specific proteins and glycan structures and contribute to adsorption to the host intestinal mucosa. The binding properties of certain SLPs are considered to exert a competitive inhibitory effect on infection because similar properties are involved in the infection mechanisms of several viruses. However, little is known regarding whether SLPs directly inhibit viral infection. In the present study, we investigated the effect of an SLP of the *Lactobacillus crispatus* KT-11 strain, a probiotic strain isolated from a healthy human infant, on human rotavirus infection. The impact of KT-11 lithium chloride extract (KT-11 LE), which contains SLP, on the infection of the P[4] genotype human rotavirus strain DS-1 was evaluated by monitoring the amplification of viral protein 6 (VP6) expression in human intestinal epithelial Caco-2 cells by quantitative reverse transcription-polymerase chain reaction assay after infection. KT-11 LE showed a significant suppressive effect on DS-1 infection in a dose-dependent manner with pre-infection treatment, whereas post-infection treatment was not effective. A 45 KDa protein isolated from KT-11 LE was investigated for homology using the BLAST database and was found to be a novel SLP. KT-11 SLP concentrate (KT-11 SLP) significantly inhibited the proliferative process of the DS-1 strain but not that of the P[8] genotype human rotavirus strain Wa. KT-11 SLP exerted significant inhibitory effect on DS-1 infection by pre-infection treatment even after digestion with gastric juice up to 2 h. Our results provided crucial evidence that SLPs from certain *Lactobacillus* strains can inhibit human rotavirus infection of intestinal epithelial cells.

## Introduction

Lactic acid bacteria, including the genus *Lactobacillus* play a crucial role in the production of fermented dairy products such as cheese, yoghurt, and fermented milk. In recent years, there has been considerable focus on the action of the S-layer protein (SLP) of lactic acid bacteria as an antiviral component. SLPs are proteinaceous subunits that exist as the outermost components of the cell walls of several genera and species of bacteria and archaea. SLPs in *Lactobacillus* strains isolated from the intestine are considered to function as adhesion molecules to the host by recognizing extracellular proteins and carbohydrates (Hynönen and Palva, 2013). Certain SLPs are anticipated to have potential inhibitory effects on viral infections due to their immunomodulatory properties and competitive binding to target molecules of infection. Furthermore, the SLPs of several *Lactobacillus* species have been reported to inhibit viral infection by activating the antiviral immune system (Gao et al., 2016). In addition, the SLP from *L. acidophilus* ATCC 4356 has been reported to specifically block Junin virus, H9N2 avian influenza virus, alphavirus, and flavivirus infections by competitively binding to dendritic cell-specific intercellular adhesion molecule 3-grabbing non-integrin (DC-SIGN) (Martínez et al., 2012; Prado Acosta et al., 2019). However, it is unclear whether the SLP is efficacious in preventing other viruses.

Rotaviruses are the leading cause of severe diarrhea in younger children worldwide, causing an estimated 453,000 deaths each year (Tate et al., 2012). A systematic analysis conducted in 2015 showed that rotavirus infections accounted for 29.3% of all diarrheal deaths in children younger than 5 years (Troeger et al., 2018). The World Health Organization recommended rotavirus vaccination in all national immunization programs in 2009; however, the total cost of vaccination remains high, especially in low- and middle-income countries (Pecenka et al., 2018). Therefore, there is an urgent need to identify and characterize effective natural materials that can be employed routinely to prevent rotavirus infections.

In the present study, we focused on the antiviral effects of the SLP of *Lactobacillus crispatus* strain KT-11, a probiotic strain isolated from a healthy infant (Tobita et al., 2009, 2010, 2018). Lithium chloride extract (LE) containing SLP (Johnson et al., 2013) was prepared from the KT-11 strain and evaluated for its inhibitory effect on human rotavirus infection in human intestinal epithelial cells.

## Materials and Methods

### Lactic Acid Bacteria

The KT-11 strain (GenBank accession number: AP025162, FERM BP-11332) was provided as a stock culture from KITII Co., Ltd. (Tokyo, Japan). KT-11 was inoculated in DeMan–Rogosa–Sharpe (MRS) broth, cultivated for 24 h at 37°C, collected by centrifugation, washed thrice with sterile water, and lyophilized.

### Virus

Human rotavirus strains DS-1 (VR-2550) and Wa (VR-2018) were purchased from the American Type Culture Collection (Manassas, VA, United States). The viruses were treated with trypsin for 1 h before use, propagated in MA104 cells, and harvested after two freeze–thaw cycles.

### Cells

The human colon carcinoma cell line Caco-2 was purchased from the Riken BioResource Research Center (Ibaraki, Japan). Cells were cultured in monolayer cultures using complete high-glucose Eagle’s minimal essential medium (EMEM; FUJIFILM Wako Pure Chemical, Osaka, Japan) containing 10% (v/v) heat-inactivated fetal bovine serum (FBS; Sigma-Aldrich, St. Louis, MO, United States), 100 U/mL penicillin G, and 100 μg/mL streptomycin (Sigma-Aldrich). The cells were passaged using TrypLE Express (Thermo Fisher Scientific, Waltham, MA, United States) and cultured under a humidified atmosphere of 5% CO_2_/95% air at 37°C.

### Preparation of KT-11 Lithium Chloride Extract and S-Layer Protein

The KT-11 strain grown in MRS medium was collected by centrifugation (8,000 × *g*) at 4°C for 5 min. The pellet was washed by centrifugation (8,000 × *g*) thrice with cold distilled water. Bacterial cell pellets were suspended in 100 mL of 5 M lithium chloride and incubated in a shaker at 37°C for 2 h. Thereafter, the suspension was centrifuged (8,000 × *g*) at 4°C for 5 min, and the supernatant was filtered through a membrane filter with 0.20 μm pore diameter (Merck Millipore, Darmstadt, Germany). The filtrate was then dialyzed against distilled water at 4°C for 24 h. The dialyzate was centrifuged at 14,000 × *g* at 4°C for 5 min, and the precipitate was lyophilized to produce KT-11 lithium chloride extract (KT-11 LE) powder. KT-11 LE was obtained at a yield of 7.8%, determined on the basis of wet weight. KT-11 SLP concentrate (KT-11 SLP) was prepared from KT-11 LE using the method described by Johnson et al. (2013). Briefly, KT-11 LE was mixed with 1 M lithium chloride at 37°C for 24 h. The insoluble fraction was collected by centrifugation and lyophilized. KT-11 LE and KT-11 SLP was dissolved in sterile distilled water for subsequent study.

### Rotavirus Infection of Target Cells

**FIGURE 1:**
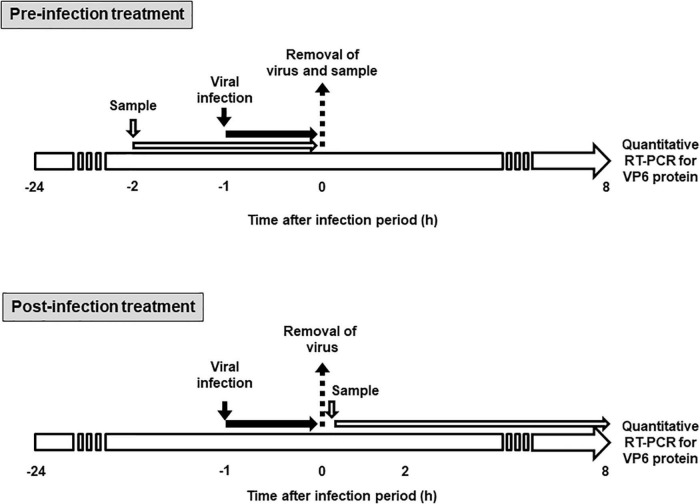
Schematic diagram for sample treatment in rotavirus infection study. In pre-infection treatment, Caco-2 cells were treated with KT-11 LE or KT-11 SLP for 1 h, then infected with the virus for 1 h, and the amplification of VP6 mRNA after 8 h was analyzed by quantitative RT-PCR. In post-infection treatment, Caco-2 cells were infected with the virus for 1 h and then treated with the KT-11 LE or KT-11 SLP for 8 h, and the amplification of VP6 mRNA was analyzed by quantitative RT-PCR.

The effect of KT-11 strain and its components on rotavirus infectivity was assessed using the method shown in Figure 1. In pre-infection treatment, Caco-2 cells were seeded at 2.0 × 10^5^ cells/mL/well in 24-well plates (Thermo Fisher Scientific, Waltham, MA, United States) and cultured at 37°C. After 24 h, the culture supernatant was discarded, and the cells were incubated with the KT-11 LE or KT-11 SLP in 200 μL of serum-free EMEM for 1 h, following which either the Wa or the DS-1 strain was added and incubated for another 1 h. The culture supernatant was removed, the cells were washed with serum-free EMEM and further cultured in complete EMEM under a humidified atmosphere of 5% CO_2_/95% air at 37°C. After 24 h, the cells were collected for quantitative reverse transcription-polymerase chain reaction (RT-PCR).

In post-infection treatment, Caco-2 cells were seeded at 2.0 × 10^5^ cells/mL/well in 24-well plates (Thermo Fisher Scientific) and cultured at 37°C. After 24 h, the culture supernatant was discarded, and Wa or DS-1 strain was added and incubated for another 1 h. Thereafter, the culture supernatant containing uninfected virus particles was removed, the cells were washed with serum-free EMEM and further cultured in complete EMEM supplemented with the KT-11 LE or KT-11 SLP under a humidified atmosphere of 5% CO_2_/95% air at 37°C. After 24 h, the cells were collected for quantitative RT-PCR.

### Quantitative Reverse Transcription-Polymerase Chain Reaction Assay

Total RNA was extracted from rotavirus-infected Caco-2 cells using TRI reagent (Sigma-Aldrich) according to the manufacturer’s protocol. The extracted RNA (1 μg) was reverse transcribed in a thermal cycler (PTC-200; MJ Research, Waltham, MA, United States) with 1 mM of each dNTP, 2.5 units/μL M-MLV reverse transcriptase (Thermo Fisher Scientific), and 10 pmol/μL of oligo(dT)18 primers at 42°C for 50 min. Quantitative RT-PCR of the resulting cDNA was performed using 0.5 μg cDNA with TB Green Premix Ex Taq II (Takara Bio, Shiga, Japan) and 10 pmol/μL primers. The primer sequences are shown in Table 1. The PCR comprised one cycle of preheating (95°C, 10 min) and 40 cycles of denaturation (95°C, 10 s) and primer annealing and extension (55°C, 30 s) using the Eco Real Time PCR System (Illumina, San Diego, CA, United States). Results were analyzed with the ΔΔCt method using the Eco system software (Illumina). The amount of PCR products was normalized to the expression level of GAPDH.

**TABLE 1:** Primer sequences used in this study.

Target	Sequence		Product size (bp)	Accession number
	Forward	Reverse		
DS-1 VP6*	5′-CCATCAATGCACCAGCCAAC-3′	5′-GCCGTCGTTAATACTCTGCG-3′	92	EF583028.1
Wa VP6*	5′-GTGAATCAGTGCTTGCGGAC-3′	5′-ATGCCTGGTGGAAATACCGG-3′	103	K02086.1
Human GAPDH*	5′-AACGGATTTGGTCGTATTGG-3′	5′-AATGAAGGGGTCATTGATGG-3′	90	BT006893.1

* VP6, viral protein 6; GAPDH, glyceraldehyde-3-phosphate dehydrogenase.

### Identification of Protein

The identification of the expressed 45 kDa protein was based on the observed molecular weights of the proteins according to Antikainen et al. (2002). Briefly, the target band of the 45 kDa protein in 12% SDS-PAGE gel was digested by trypsin, followed by analysis with liquid chromatography-tandem mass spectrometry (LC-MS/MS). The generated data were subjected to a search using the Mascot (Matrix Science, London, United Kingdom) algorithm. Protein identification was carried out using all the SLP sequences of KT-11. Homology searches were performed using the BLAST program (available at^1^). Sequences of eight *Lactobacillus* SLP sequences were aligned using CLUSTALW^2^. The GenBank accession numbers for the SLP sequences were as follows: AAB58734 for CbsA of *L. crispatus* JCM 5810; AFB69875 for SlpA of *L. crispatus* K313; ABI49163 for SlpA of *L. crispatus* ZJ001: CAA61560 for SlpA of *L. acidophilus* JCM 4356; YP_193101 for SlpA from *L. acidophilus* NCFM; ATO53009 for the SLP of *L. amylovorus* JCM1126T; and CAB46985 for the SLP of *L. helveticus* JCM1120T. Both amino acid sequencing and peptide mapping were performed by Japan Proteomics Co., Ltd. (Miyagi, Japan).

### Enzymatic Digestion

Simulated digestive juice was prepared as gastric juice containing 1.6% pepsin (Sigma-Aldrich) in 0.1 M KCl/HCl (pH 2.0) and intestinal juice containing 0.1% pancreatin (Sigma-Aldrich) in PBS (pH 7.2). KT-11 LE and KT-11 SLP were treated with gastric juice or intestinal juice for the indicated time periods at 37°C. The sensitivity of KT-11 SLP to digestive enzyme treatment was evaluated by sodium dodecyl sulphate-polyacrylamide gel electrophoresis (SDS-PAGE) using a discontinuous buffer system with 12.5% separating gel and 5% stacking gel. Protein bands were visualized by staining the gels with Quick-CBB (FUJIFILM Wako Pure Chemical).

To determine the inhibitory activity of SLP after gastric digestion, SLP was dissolved in gastric juice and incubated at 37°C. After treatment for 0, 0.5, 1, 2, and 3 h, the SLP solution was neutralized with NaOH to adjust pH to 7.2, and was subjected to the pre-infection treatment assay.

### Statistical Analysis

The data were statistically analyzed using one-way analysis of variance (ANOVA) with Tukey–Kramer multiple comparison tests using Excel 2019 (Microsoft, Redmond, WA, United States) with the add-in software Statcel4 (OMS Publishing, Tokyo, Japan). Results with a *p*-value of less than 0.05 were considered statistically significant.

## Results

### Effect of KT-11 Lithium Chloride Extract on the Infection of DS-1 Strain in Caco-2 Cells

**FIGURE 2:**
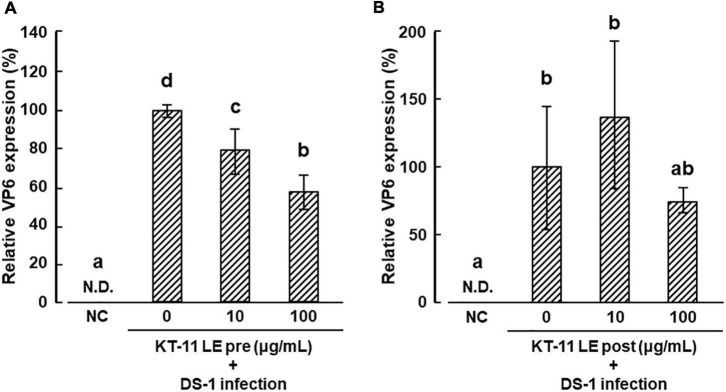
Effect of KT-11 LE against rotavirus infection in Caco-2 cells. **(A)** Caco-2 cells were treated with KT-11 LE at the indicated concentration 1 h prior to DS-1 infection. The expression of VP6 in Caco-2 cells 24 h after the infection was evaluated. The results are shown as means with standard deviations relative to KT-11 LE-untreated control (*n* = 3). N.D. denotes not detected. Different letters denote a significant difference at *p* < 0.05. **(B)** Caco-2 cells were treated with KT-11 LE at the indicated concentration after DS-1 infection. The expression of VP6 in Caco-2 cells 24 h after the infection was evaluated. The results are shown as means with standard deviations relative to KT-11 LE-untreated and viral-infected control (*n* = 3). NC represents the condition of viral-uninfected (and also KT-11 LE-untreated) condition. N.D. denotes not detected.

The effect of KT-11 LE on the infection of DS-1 human rotavirus in Caco-2 cells is shown in Figures 2A,B. In the pre-infection treatment, KT-11 LE significantly suppressed the amplification of VP6 mRNA in a dose-dependent manner (Figure 2A). In contrast, in the post-infection treatment, no significant suppression of DS-1 strain infection was observed with KT-11 LE at all tested concentrations (Figure 2B).

### Identification of the 45 kDa Protein

**FIGURE 3:**
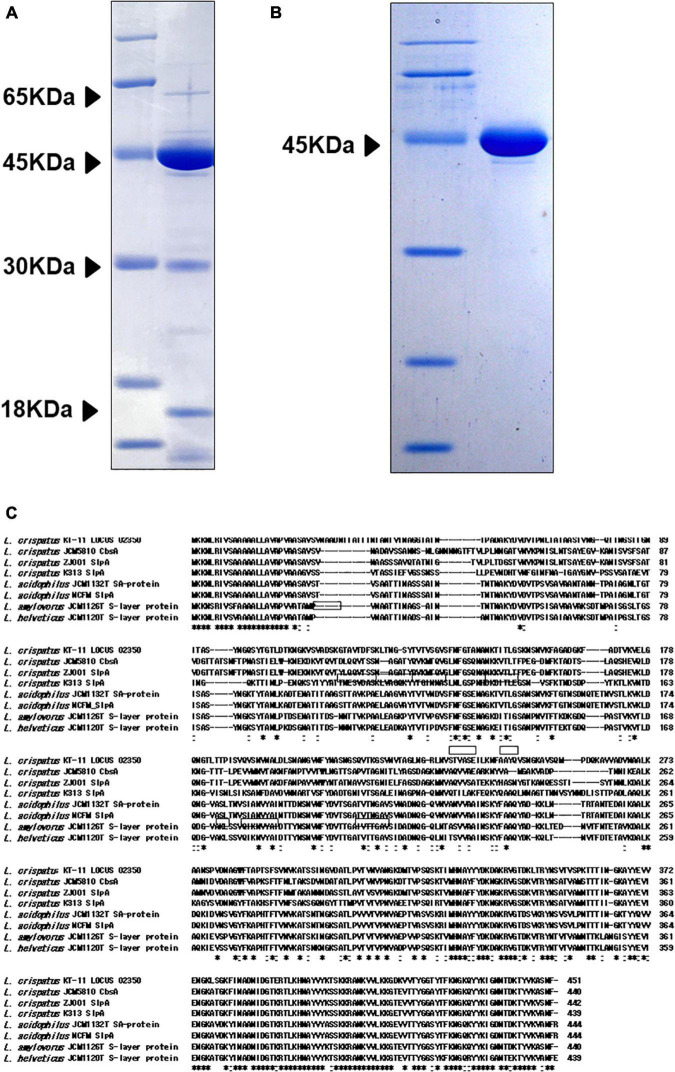
SDS-PAGE pattern and amino acid sequence of the 45 kDa protein. SDS-PAGE patterns of **(A)** KT-11 LE and concentrated 45 kDa protein **(B)**. **(C)** Comparison of the revealed amino acid sequences of the 45 kDa protein and SLPs from other *Lactobacillus* strains. The framed areas show the amino acid sequences revealed by protein identification analysis using NanoLC-MS/MS. Hyphens indicate deletions or insertions of amino acids. “*” denotes that the residues are identical in all sequences in the alignment, and “:” denotes that conserved substitutions are observed.

Sodium dodecyl sulfate-polyacrylamide gel electrophoresis analysis of KT-11 LE revealed the presence of 18, 30, 45, and 65 kDa proteins as its major proteinaceous components (Figure 3A). Among them, the 45 kDa protein was concentrated as an insoluble fraction in 1 M lithium chloride (Figure 3B).

The nine partial amino acid sequences of the 45 kDa peptide identified by LC-MS/MS were GTAVTDFSK, GSVNVTAGLNGR, LNVSTVASEILK, NFAAYQVSNGK, AVVADVNAALK, YNSVTVSPK, AYYEVVENGK, FINADNIDGTER, and VVTYGGTYTFK. Mascot search of these results revealed that all the sequences were included in the translated product of the SLP gene (LOCUS_02350) of KT-11 (Figure 3C). Comparing this amino acid sequence with SLPs from other *Lactobacillus* strains revealed that the 45 kDa protein was a novel SLP with significant homology in the C-terminal region and notable difference in the *N*-terminal and middle regions.

### Effect of KT-11 S-Layer Protein on DS-1 and Wa Strain Infection in Caco-2 Cells

**FIGURE 4:**
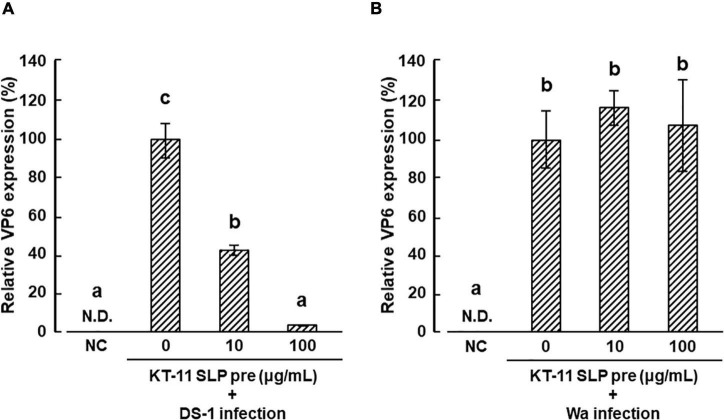
Effect of KT-11 SLP against rotavirus infection in Caco-2 cells. Caco-2 cells were treated with KT-11 SLP at the indicated concentrations 1 h prior to DS-1 **(A)** or Wa **(B)** strain infection. The expression of VP6 in Caco-2 cells 24 h after infection was evaluated. The results are shown as means with standard deviations relative to KT-11 SLP-untreated and viral-infected control (*n* = 3). NC represents the condition of viral-uninfected (and also KT-11 SLP-untreated) condition. N.D. denotes not detected. Different letters denote a significant difference at *p* < 0.05.

The effect of KT-11 SLP on the infection of DS-1 strain in Caco-2 cells is shown in Figure 4A. DS-1 infection was significantly suppressed by pre-infection treatment with KT-11 SLP in a concentration-dependent manner. Conversely, KT-11 SLP did not suppress the infection of the Wa strain even after pre-infection treatment at 100 μg/mL (Figure 4B).

### Digestive Juice Tolerance of the Protein Component of KT-11 Lithium Chloride Extract

**FIGURE 5:**
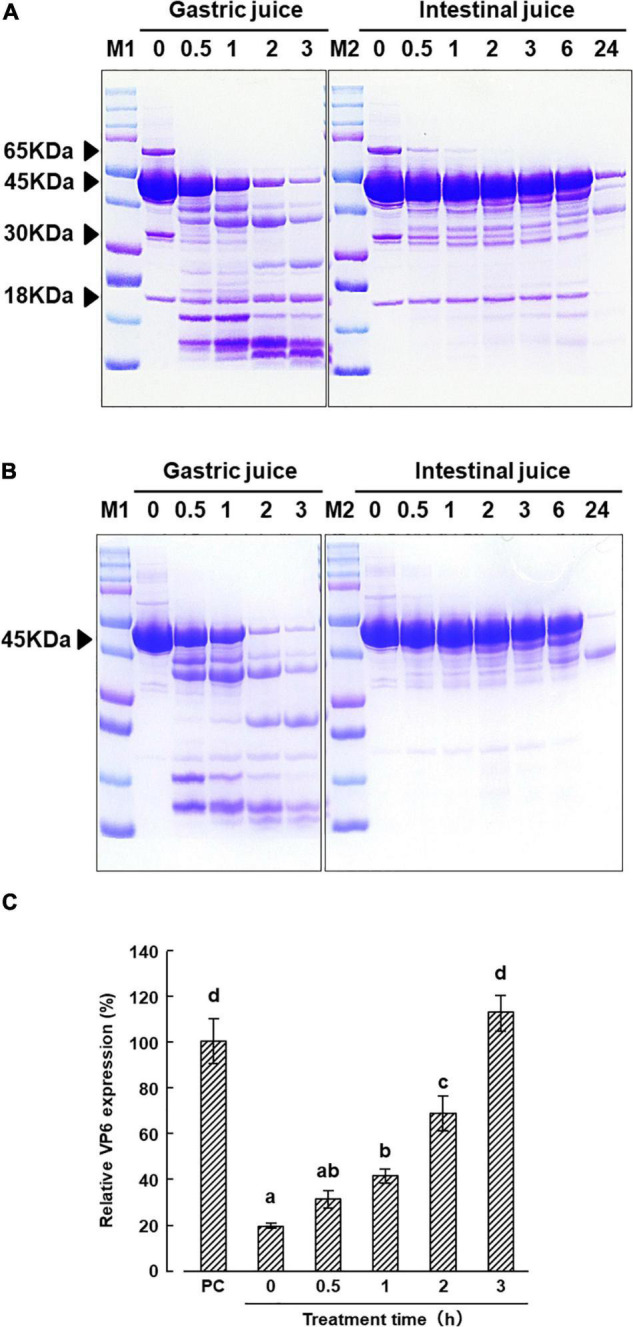
Effect of digestive juice treatment on KT-11 LE and KT-11 SLP. SDS-PAGE patterns of **(A)** KT-11 LE and fractionated LE, **(B)** KT-11 SLP after treatment with simulated gastric juice (1.6% pepsin in 0.1 M KCl/HCl, pH 2.0) and intestinal juice (0.1% pancreatin in PBS, pH 7.2) for the indicated time periods. Lane 1 (M1) and lane 7 (M2): molecular weight marker, lanes 2–6: digestion pattern by gastric juice for the indicated time periods (h), lanes 8–14: digestion pattern by intestinal juice for the indicated time periods (h). **(C)** Inhibitory effect on DS-1 infection in pre-infection treatment with KT-11 SLP digested with gastric juice for the indicated times periods. The results are shown as means with standard deviations relative to KT-11 SLP-untreated viral-infected control (*n* = 3). PC represents the condition of viral-infected and KT-11 SLP-untreated condition. Different letters denote a significant difference at *p* < 0.01.

Sodium dodecyl sulfate-polyacrylamide gel electrophoresis patterns of KT-11 LE and the KT-11 SLP after exposure to simulated digestive juice are shown in Figures 5A,B. The 45 kDa protein band remained visible up to 3 h after the addition of simulated gastric juice, though it gradually disappeared with prolonged incubation. Only a slight decrease in the intensity of the 45 kDa protein band was observed till 6 h after the addition of simulated intestinal juice, while a more substantial decrease was observed after 24 h. Conversely, the bands of 18, 30, and 65 kDa proteins in KT-11 LE completely disappeared following the 24-h treatment with intestinal juice.

From 0 to 2 h after treatment with gastric juice, the inhibitory effect of KT-11 SLP on DS-1 strain infection in the pre-infection treatment decreased in a treatment time dependent manner. Significant inhibition was observed even after 2 h, but the activity was completely lost after 3 h digestion of KT-11 SLP with gastric juice (Figure 5C).

## Discussion

In the present study, we found that KT-11 LE inhibited infection of the intestinal cells by the human rotavirus DS-1 strain and confirmed that the 45 kDa protein, identified by SDS-PAGE, was one of the active components of KT-11 LE. Although SLPs have also been reported from other strains of *Lactobacillus*, the binding targets of most SLPs remain unelucidated. Sillanpää et al. (2000) and Sun et al. (2013) reported that CbsA from *L. crispatus* JCM5810 and SlpB from *L. crispatus* K313 bind to type I collagen and type IV collagen, respectively. Konstantinov et al. (2008) and the above-mentioned study by Martínez et al. (2012) showed that the binding target of SlpA from *L. acidophilus* NCFM and SLP from *L. acidophilus* ATCC 4356 is DC-SIGN. The inhibition of DS-1 infection only after pre-infection treatment but not post-infection treatment with KT-11 LE (Figure 2) implied that there may be an underlying mechanism of inhibition by competitive binding to the target molecules. The 45 kDa protein identified herein as KT-11 SLP may bind to the same target molecule of these known SLPs from closely related *Lactobacillus* species because it is homologous to their amino acid sequence (Figure 3C); however, neither target molecule has been reported as a receptor for human rotavirus.

The entry of rotavirus into cells is a complex multistep process, in which different domains of rotavirus surface proteins interact with cell-surface molecules that function as receptors for adhesion and entry (López and Arias, 2004, 2006). Among them, several carbohydrates, such as terminal sialic acids (Isa et al., 2006; Haselhorst et al., 2009) and histo-blood group antigens (HBGA) (Tan and Jiang, 2014; Jiang et al., 2017), have been reported to be involved in rotavirus attachment to target cells. As shown in Figure 4A, KT-11 SLP significantly inhibited the infection of the DS-1 strain in Caco-2 cells in a dose-dependent manner. The initial interactions of human rotavirus strains with host cells is dependent on the VP4 genotype (Ciarlet et al., 2002). According to the classification based on the molecular properties of VP4 (P-types), the DS-1 strain is classified as the P[4] genotype (Matthijnssens et al., 2008). Increasing evidence indicates that the P[4] genotype rotaviruses, including the DS-1 strain, use H-type 1 and Lewis-b antigens for infection (Huang et al., 2012; Ramani et al., 2016). H-type 1 and Lewis-b antigens have been reported in Caco-2 cells (Amano and Oshima, 1999; Murakami et al., 2013). Our results suggest that the possibility of that DS-1 infection is inhibited by competitive binding of KT-11 SLP to these antigens. However, contrary to our expectations, infection of the Wa strain, another dominant P[8] subtype reported using the same H-type antigen and Lewis-b for infection (Huang et al., 2012; Ramani et al., 2016), was not inhibited in the presence of KT-11 SLP (Figure 4B).

Although it is unclear why KT-11 SLP failed to inhibit infection of the Wa strain, the simplest possibility is that KT-11 SLP binds to a molecule used only by the DS-1 strain. However, we could not name a specific candidate molecule to explain this hypothesis; the A-type antigen reported by Böhm et al. (2015) is a possible candidate molecule, but is not expressed in Caco-2 cells derived from O-type patients. Another possible reason is that KT-11 SLP binds to common targets of DS-1 and Wa strains, such as H-type 1 and Lewis-b antigens, but may not exert its inhibitory effect due to the presence of ganglioside GM1, which has been reported to bind only to Wa strains (Ramani et al., 2016). Recently, Laucirica et al. (2017) reported that DS-1 strain infection was significantly inhibited by 3′-sialyl lactose and 6′-sialyl lactose, whereas the Wa strain was inhibited by 2′-fucosyl lactose, implying that compounds containing sialic acid may be deeply involved in the infection of DS-1 strain. In support of the involvement of sialic acid in the inhibitory effect of KT-11 SLP on human rotaviruses, we found that KT-11 SLP agglutinated sheep erythrocytes, the aggregation of which by viruses targeting sialic acid is widely known (Supplementary Figure 1). Furthermore, in preliminary studies of this study, we have confirmed the significant diarrhea-preventive effect of oral administration of KT-11 LE in an *in vivo* diarrheal model caused by the monkey rotavirus SA-11 virus known for sialic acid-dependent binding (Supplementary Figure 2), as well as the effect of KT-11 SLP on A similar inhibitory effect has been observed in an *in vitro* infection system of SA-11 strains (Supplementary Figure 3). Although the target molecule of the hemagglutinating reaction and inhibition of SA-11 infection by KT-11 SLP is not clear at present, based on the above results, we predict that DS-1 strain may be more dependent on binding to sialic acid-related structures than that of Wa strain, which may have led to the difference in inhibitory effect in this experiment. Further studies are needed to elucidate the inhibitory effect of KT-11 SLP on human rotavirus infection.

In conclusion, the present study demonstrated that KT-11 LE and its component, a novel SLP, inhibited the infection of the human rotavirus DS-1 strain. As KT-11 SLP showed some degree of resistance to digestion with gastric juice (Figure 5), it may work to prevent rotavirus infection in the intestine by withstanding digestion, particularly in infants with low stomach pH and limited action of pepsin (Nguyen et al., 2015; Neal-Kluever et al., 2019). Rotavirus infection is a serious problem in infants with a compromised immune system. Although further experimental studies are needed to fully understand the mechanism by which SLPs prevent rotavirus infection *in vivo*, the obtained findings suggested that SLPs derived from specific strains of lactic acid bacteria may be used as drugs or food additives to prevent or ameliorate conditions caused by rotavirus, especially in infants and young children.

## Data Availability Statement

The original contributions presented in this study are included in the article/Supplementary Material, further inquiries can be directed to the corresponding author.

## Author Contributions

TK: conception and design, drafting the work or revising it critically for important intellectual content, and writing the manuscript. TK, IS, and YT: acquisition of data. TK, KT, MT, and IW: interpretation of results. All authors contributed to the article and approved the submitted version.

## Conflict of Interest

TK, MT, and IW were employed by KITII Co., Ltd. The remaining authors declare that the research was conducted in the absence of any commercial or financial relationships that could be construed as a potential conflict of interest.

## Publisher’s Note

All claims expressed in this article are solely those of the authors and do not necessarily represent those of their affiliated organizations, or those of the publisher, the editors and the reviewers. Any product that may be evaluated in this article, or claim that may be made by its manufacturer, is not guaranteed or endorsed by the publisher.

## Footnotes

1
http://www.ncbi.nlm.nih.gov/BLAST/

2
http://www.ebi.ac.uk/clustalw/

## Abbreviations

SLP: S-layer protein
LE: lithium chloride extract
MRS: DeMan–Rogosa–Sharpe
EMEM: Eagle’s minimal essential medium
FBS: fetal bovine serum
RT-PCR: reverse transcription-polymerase chain reaction
VP6: viral protein 6
GAPDH: glyceraldehyde-3-phosphate dehydrogenase
LC-MS/MS: liquid chromatography-tandem mass spectrometry
SDS-PAGE: sodium dodecyl sulfate-polyacrylamide gel electrophoresis
ANOVA: analysis of variance
DC-SIGN: dendritic cell-specific intercellular adhesion molecule 3-grabbing non-integrin
HBGA: human blood group antigen.

## References

[B1] Amano J. Oshima M. (1999). Expression of the H type 1 blood group antigen during enterocytic differentiation of Caco-2 cells. *J. Biol. Chem.* 274 21209–21216. 10.1074/jbc.274.30.21209 10409676

[B2] Antikainen J. Anton L. Sillanpää J. Korhonen T. K. (2002). Domains in the S-layer protein CbsA of *Lactobacillus crispatus* involved in adherence to collagens, laminin and lipoteichoic acids and in self-assembly. *Mol. Microbiol.* 46 381–394. 10.1046/j.1365-2958.2002.03180.x 12406216

[B3] Böhm R. Fleming F. E. Maggioni A. Dang V. T. Holloway G. Coulson B. S. (2015). Revisiting the role of histo-blood group antigens in rotavirus host-cell invasion. *Nat. Commun.* 6:5907. 10.1038/ncomms6907 25556995

[B4] Ciarlet M. Ludert J. E. Iturriza-Gómara M. Liprandi F. Gray J. J. Desselberger U. (2002). Initial interaction of rotavirus strains with *N*-acetylneuraminic (sialic) acid residues on the cell surface correlates with VP4 genotype, not species of origin. *J. Virol.* 76 4087–4095. 10.1128/JVI.76.8.4087-4095.2002 11907248 PMC136071

[B5] Gao X. Huang L. Zhu L. Mou C. Hodrddu Q. Yu Q. (2016). Inhibition of H9N2 Virus invasion into dendritic cells by the S-layer protein from *L. acidophilus* ATCC 4356. *Front. Cell. Infect. Microbiol.* 6:137. 10.3389/fcimb.2016.00137 27826541 PMC5078685

[B6] Haselhorst T. Fleming F. E. Dyason J. C. Hartnell R. D. Yu X. Holloway G. (2009). Sialic acid dependence in rotavirus host cell invasion. *Nat. Chem. Biol.* 5 91–93. 10.1038/nchembio.134 19109595

[B7] Huang P. Xia M. Tan M. Zhong W. Wei C. Wang L. (2012). Spike protein VP8* of human rotavirus recognizes histo-blood group antigens in a type-specific manner. *J. Virol.* 86 4833–4843. 10.1128/JVI.05507-11 22345472 PMC3347384

[B8] Hynönen U. Palva A. (2013). *Lactobacillus* surface layer proteins: structure, function and applications. *Appl. Microbiol. Biotechnol.* 97 5225–5243. 10.1007/s00253-013-4962-2 23677442 PMC3666127

[B9] Isa P. Arias C. F. López S. (2006). Role of sialic acids in rotavirus infection. *Glycoconj. J.* 23 27–37. 10.1007/s10719-006-5435-y 16575520 PMC7087688

[B10] Jiang X. Liu Y. Tan M. (2017). Histo-blood group antigens as receptors for rotavirus, new understanding on rotavirus epidemiology and vaccine strategy. *Emerg. Microbes Infect.* 6:e22. 10.1038/emi.2017.30 28400594 PMC5457676

[B11] Johnson B. Selle K. O’Flaherty S. Goh Y. J. Klaenhammer T. (2013). Identification of extracellular surface-layer associated proteins in *Lactobacillus acidophilus* NCFM. *Microbiology* 159 2269–2282. 10.1099/mic.0.070755-0 24002751 PMC3836491

[B12] Konstantinov S. R. Smidt H. de Vos W. M. Bruijns S. C. Singh S. K. Valence F. (2008). S layer protein A of *Lactobacillus acidophilus* NCFM regulates immature dendritic cell and T cell functions. *Proc. Natl. Acad. Sci. U.S.A.* 105 19474–19479. 10.1073/pnas.0810305105 19047644 PMC2592362

[B13] Laucirica D. R. Triantis V. Schoemaker R. Estes M. K. Ramani S. (2017). Milk oligosaccharides inhibit human rotavirus infectivity in MA104 cells. *J. Nutr.* 147 1709–1714. 10.3945/jn.116.246090 28637685 PMC5572490

[B14] López S. Arias C. F. (2004). Multistep entry of rotavirus into cells: a Versaillesque dance. *Trends Microbiol.* 12 271–278. 10.1016/j.tim.2004.04.003 15165605

[B15] López S. Arias C. F. (2006). Early steps in rotavirus cell entry. *Curr. Top. Microbiol. Immunol.* 309 39–66. 10.1007/3-540-30773-7_2 16909896

[B16] Martínez M. G. Prado Acosta M. Candurra N. A. Ruzal S. M. (2012). S-layer proteins of *Lactobacillus acidophilus* inhibits JUNV infection. *Biochem. Biophys. Res. Commun.* 422 590–595. 10.1016/j.bbrc.2012.05.031 22595457 PMC7124250

[B17] Matthijnssens J. Ciarlet M. Rahman M. Attoui H. Bányai K. Estes M. K. (2008). Recommendations for the classification of group A rotaviruses using all 11 genomic RNA segments. *Arch. Virol.* 153 1621–1629. 10.1007/s00705-008-0155-1 18604469 PMC2556306

[B18] Murakami K. Kurihara C. Oka T. Shimoike T. Fujii Y. Takai-Todaka R. (2013). Norovirus binding to intestinal epithelial cells is independent of histo-blood group antigens. *PLoS One* 8:e66534. 10.1371/journal.pone.0066534 23799113 PMC3682964

[B19] Neal-Kluever A. Fisher J. Grylack L. Kakiuchi-Kiyota S. Halpern W. (2019). Physiology of the neonatal gastrointestinal system relevant to the disposition of orally administered medications. *Drug Metab. Dispos.* 47 296–313. 10.1124/dmd.118.084418 30567878

[B20] Nguyen T. T. P. Bhandari B. Cichero J. Prakash S. (2015). A comprehensive review on *in vitro* digestion of infant formula. *Food Res. Int.* 76 373–386. 10.1016/j.foodres.2015.07.016 28455017

[B21] Pecenka C. Debellut F. Bar-Zeev N. Anwari P. Nonvignon J. Shamsuzzaman M. (2018). Re-evaluating the cost and cost-effectiveness of rotavirus vaccination in Bangladesh, Ghana, and Malawi: a comparison of three rotavirus vaccines. *Vaccine* 36 7472–7478. 10.1016/j.vaccine.2018.10.068 30420039 PMC6238205

[B22] Prado Acosta M. Geoghegan E. M. Lepenies B. Ruzal S. Kielian M. Guadalupe Martinez M. (2019). Surface (S) layer proteins of *Lactobacillus acidophilus* block virus infection *via* DC-SIGN interaction. *Front. Microbiol.* 2019:810. 10.3389/fmicb.2019.00810 31040840 PMC6477042

[B23] Ramani S. Hu L. Venkataram Prasad B. V. Estes M. K. (2016). Diversity in rotavirus-host glycan interactions: a “sweet” spectrum. *Cell. Mol. Gastroenterol. Hepatol.* 2 263–273. 10.1016/j.jcmgh.2016.03.002 28090561 PMC5042371

[B24] Sillanpää J. Martínez B. Antikainen J. Toba T. Kalkkinen N. Tankka S. (2000). Characterization of the collagen-binding S-layer protein CbsA of *Lactobacillus crispatus*. *J. Bacteriol.* 182 6440–6450. 10.1128/JB.182.22.6440-6450.2000 11053389 PMC94791

[B25] Sun Z. Kong J. Hu S. Kong W. Lu W. Liu W. (2013). Characterization of a S-layer protein from *Lactobacillus crispatus* K313 and the domains responsible for binding to cell wall and adherence to collagen. *Appl. Microbiol. Biotechnol.* 97 1941–1952. 10.1007/s00253-012-4044-x 22526799

[B26] Tan M. Jiang X. (2014). Histo-blood group antigens: a common niche for norovirus and rotavirus. *Expert Rev. Mol. Med.* 16:e5. 10.1017/erm.2014.2 24606759 PMC12406300

[B27] Tate J. E. Burton A. H. Boschi-Pinto C. Steele A. D. Duque J. Parashar U. D. (2012). 2008 estimate of worldwide rotavirus-associated mortality in children younger than 5 years before the introduction of universal rotavirus vaccination programmes: a systematic review and meta-analysis. *Lancet Infect. Dis.* 12 136–141. 10.1016/S1473-3099(11)70253-5 22030330

[B28] Tobita K. Watanabe I. Tomokiyo M. Saito M. (2018). Effects of heat-treated *Lactobacillus crispatus* KT-11 strain consumption on improvement of oral cavity environment: a randomised double-blind clinical trial. *Benef. Microbes* 9 585–592. 10.3920/BM2017.0137 29633644

[B29] Tobita K. Yanaka H. Otani H. (2009). Heat-treated *Lactobacillus crispatus* KT strains reduce allergic symptoms in mice. *J. Agric. Food Chem.* 57 5586–5590. 10.1021/jf900703q 19469537

[B30] Tobita K. Yanaka H. Otani H. (2010). Anti-allergic effects of *Lactobacillus crispatus* KT-11 strain on ovalbumin-sensitized BALB/c mice. *Anim. Sci. J.* 81 699–705. 10.1111/j.1740-0929.2010.00795.x 21108691

[B31] Troeger C. Khalil I. A. Rao P. C. Cao S. Blacker B. F. Ahmed T. (2018). Rotavirus vaccination and the global burden of rotavirus diarrhea among children younger than 5 years. *JAMA Pediatr.* 172 958–965. 10.1001/jamapediatrics.2018.1960 30105384 PMC6233802

