# Supplementary material for: Lactobacillus crispatus Strain KT-11 S-Layer Protein Inhibits Rotavirus Infection
Source: Front Microbiol. 2022 Feb 22;13:783879. doi: 10.3389/fmicb.2022.783879 (PMC8902352; doi:10.3389/fmicb.2022.783879)
Supplement: Supplementary file 1 [file Data_Sheet_1.PDF]

Supplementary Material

1 Materials and Methods

1.1 Hemagglutination assay

Sheep erythrocytes (Bizcom Japan, Tokyo, Japan) were washed twice with sterile phosphate buffered saline (PBS, pH 7.4), and 4% (v/v) erythrocyte suspension was prepared in PBS. For the hemagglutination assay, 25 μL of 4% erythrocyte suspension was mixed with 25 μL of 2-fold serial dilutions of a 15,000 μg/mL solution of KT-11 strain, KT-11 LE or its SLP component in 96-well titer plates (Thermo Fisher Scientific), and incubated at 37°C for 90 min. The hemagglutination titer was represented as the value of the lowest dilution factor showing hemagglutination.

1.2 Induction of diarrhea

Specific pathogen-free pregnant BALB/c mice aged 6 weeks were purchased from Japan SLC (Shizuoka, Japan) and housed at 23°C ± 3°C under a 12-h light/dark cycle. Mice were housed one per cage prior to giving birth. All animal protocols were approved by the Committee for Animal Experiments of Shinshu University.

Simian RV strain SA11 (group A, type III) was kindly supplied by Dr. Yasui in the Kiso Town Resources Institute. The Effects of KT-11 SLP on rotavirus-induced diarrhea were evaluated according to the modified method described previously [19]. Briefly, pregnant mice were separately housed and fed with the MM-3 diet (Funabashi Farm, Chiba) until they gave birth. Seven days after birth, the mice were divided into 4 groups consisting of 1 parent mouse and 9 pups each. The pups were infected with rotavirus by oral administration of 50 μL of the SA11 strain suspension mixed with 50 μL of 1 mg/mL KT-11 LE dissolved in distilled water or water alone. The incidence of diarrhea in pups was determined by checking stool consistency for 7 consecutive days. Stool consistency was scored on a 2-point scale (0, normal solid with black color; 1, loose or liquid with light brown color), and calculated cumulative incidence rate. Mice demonstrating level 1 stool were considered positive for diarrhea.

1.3 Rotavirus infection to target cells

To examine the effects of KT-11 LE on the amplification of rotavirus mRNA, rotavirus infection model using Caco-2 cells were employed. Briefly, Caco-2 cells were seeded at 2.0 × 105 cells/mL/well in 24-well plates (BD Falcon, Corning, Corning, NY, USA) and cultured at 37°C for 24 h. After 24 h, the culture supernatant was discarded and 200 μL suspension of SA11 strain was added in E-MEM for 1 h. Then virus suspension was removed, washed with twice with E-MEM, and further cultured at 37°C. After 8 h, the cells were collected for RT-PCR assay. The primer sequences for SA-11 strain VP6 [GenBank: L15384.1] was designed as 5′-CTTCTACCAGACGCGGAAAG-3′ (forward) and 5′-ATTCGGCCTGAGAATCACTG-3′ (reverse), and were complementary to 696–715 and 794–775, respectively.

2 Results

2.1 Hemagglutination activity of KT-11 strain, KT-11 LE, and KT-11 SLP

KT-11 strain, KT-11 LE, and KT-11 SLP exhibited agglutination against sheep erythrocytes (Supplemental figure 1). KT-11 SLP exhibited stronger hemagglutinating activity than KT-11 strain and KT-11 LE. The hemagglutination titer of KT-11 SLP was estimated to be 469 µg/mL.

2.2 Effect of KT-11 LE on the incidence of RV-induced diarrhea

Mouse pups inoculated with the SA11 strain started to develop diarrhea 1 day after the inoculation, which persisted to the termination of the experiment. In the control group, all mice developed diarrhea 5 days after inoculation. In contrast, 71.8% of mice inoculated with KT-11 LE showed diarrhea. From 3 to 5 days after inoculation, the incidence of diarrhea in the KT-11 LE-treated group was significantly ($P < 0.05$) lower than in the control group (Supplemental Figure 2).

2.3 Effect of KT-11 SLP on the expression of viral proteins in SA11-infected Caco-2 cells

The amplification of viral protein VP6 was observed by quantitative PCR analysis after 8h of infection in SA-11-infected Caco-2 cells. KT-11 SLP at the concentration of 10 and 100 µg/mL significantly inhibited the amplification of VP6 in a dose-dependent manner (Supplemental Figure 3).

3 Supplementary Figures

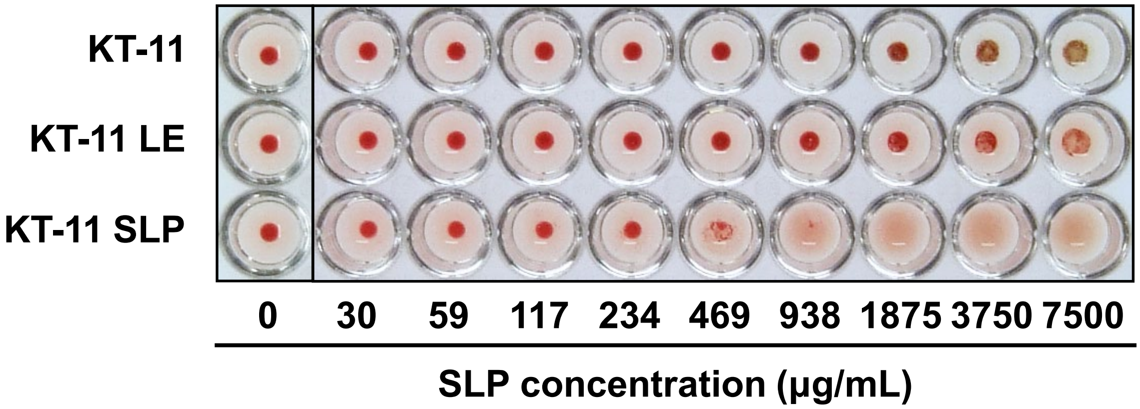

Supplementary Figure 1. Hemagglutinating activity of KT-11 strain, KT-11 LE and KT-11 SLP.

Sheep erythrocytes were mixed with 2-fold serial dilutions of KT-11 strain, KT-11 LE and KT-11 SLP at 37°C for 90 min.

Supplemental figure 2

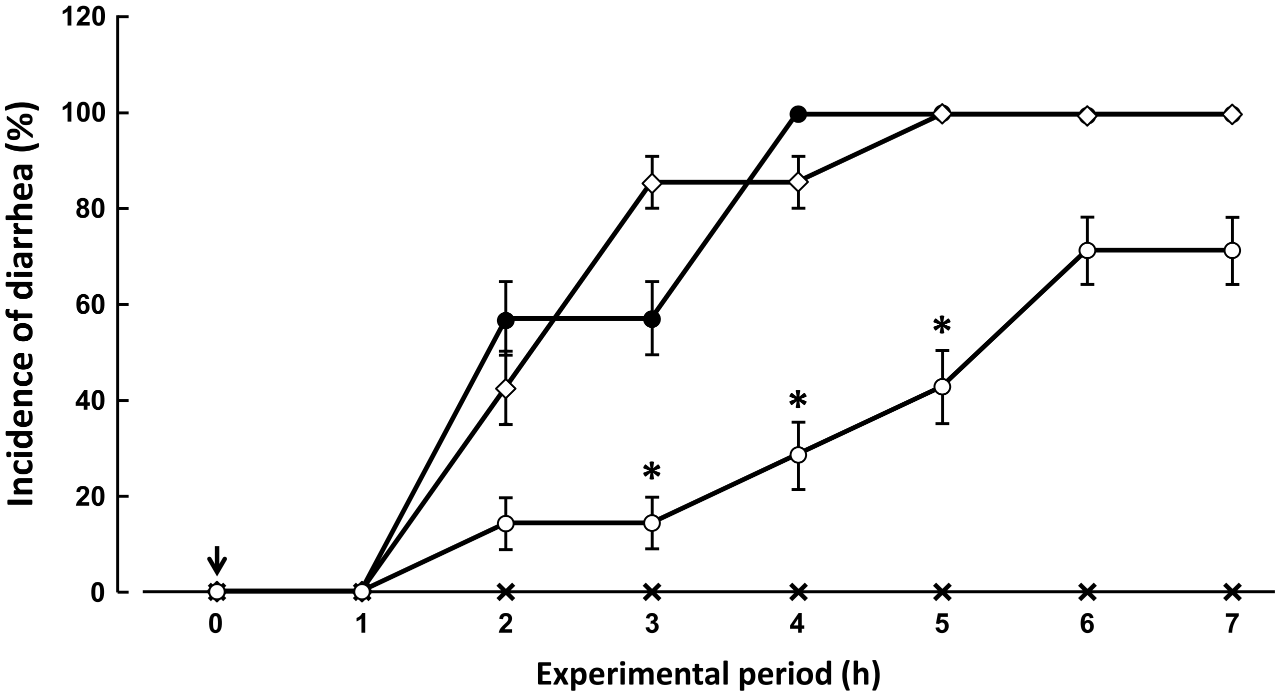

Supplemental Figure 2. The effect of KT-11 LE on the incidence of rotavirus-induced diarrhea.

Rotavirus strain SA11 and samples were simultaneously inoculated orally to neonatal BALB/c mouse pups. The incidence of diarrhea of control mice was evaluated for 7 consecutive days. Cross (×), rhombus (◇), black circle (●), and white circle (○) represent the result of group inoculated water (negative control), SA-11 + water (positive control), SA-11 + KT-11, and SA-11 + KT-11 LE, respectively. Arrow represents the time point of KT-11 LE and SA-11 inoculation. SA-11 alone (positive control), and KT-11 LE + SA-11, respectively. Data are presented as mean ± SD, (n = 9 per group). *$p < 0.05$ vs. positive control group.

Supplemental figure 3

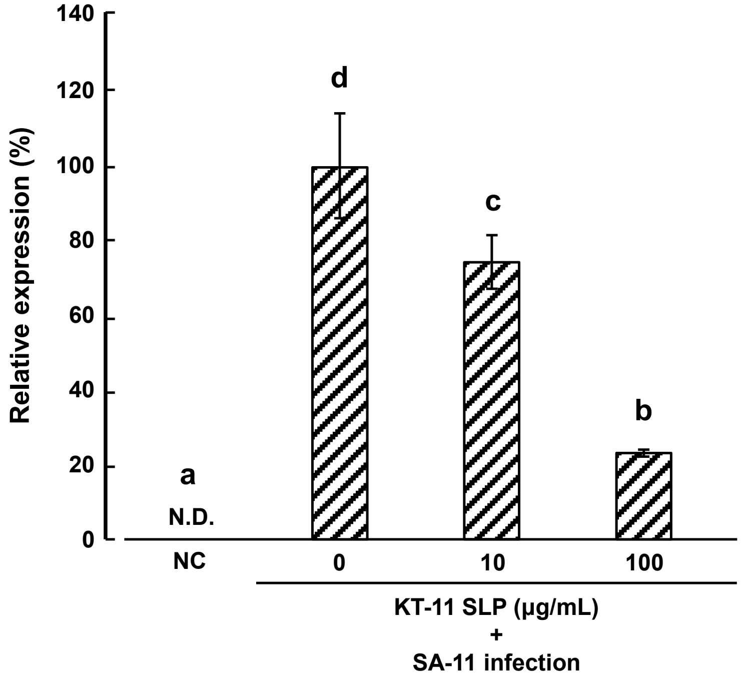

Supplemental Figure 3. The effect of KT-11 SLP against SA-11 infection in Caco-2 cells

Caco-2 cells were treated with KT-11 SLP at the indicated concentration for 24 h and then infected with the SA-11 strain for 1 h. The expression of VP6 in Caco-2 cells 8 h after infection was evaluated. The results are shown as means with standard deviations relative to KT-11 SLP-untreated and viral-infected control (n = 3). NC represents the condition of viral-uninfected (and also KT-11 SLP-untreated) condition. N.D. denotes not detected. Different letters denote a significant difference at $p < 0.05$.
